# The Sublethal Effects of β-Ecdysterone, a Highly Active Compound from *Achyranthes bidentata* Blume, on Grape Phylloxera, *Daktulosphaira vitifoliae* Fitch

**DOI:** 10.1371/journal.pone.0165860

**Published:** 2016-11-08

**Authors:** Yongqiang Liu, Fuqian Yang, Hongtie Pu, Junping Su, Zongjiang Liu, Khalid Hussain Dhiloo, Zhongyue Wang

**Affiliations:** 1 State Key Laboratory for Biology of Plant Diseases and Insect Pests, Institute of Plant Protection, Chinese Academy of Agricultural Sciences, Beijing, P. R. China; 2 Plant Protection Station of Zhongfang county, Hunan Province, Zhongfang, P. R. China; 3 Department of Entomology, Faculty of Crop Protection, Sindh Agriculture University, Tandojam, Pakistan; Universidade Federal de Vicosa, BRAZIL

## Abstract

Grape phylloxera, *Daktulosphaira vitifoliae* (Fitch) (Hemiptera, Phylloxeridae), is a very destructive insect pest of grapevines. Intercropping of *Achyranthes bidentata* Blume (f. Amaranthaceae) and *Vitis* spp. grapevines can be useful to control this pest. In the present study, the toxicity of 22 compounds, known to be present in *A*. *bidentata*, to grape phylloxera was evaluated. All treatments were toxic towards grape phylloxera but the degree of toxicity differed between treatments. Among the 22 tested compounds, several of which proved toxic towards grape phylloxera. However β-ecdysterone had higher toxic effects against grape phylloxera, with LC_50_ values of 175.73 mg a.i. liter^-1^. In addition, we assessed the sublethal effects of LC_10_, LC_20_ and LC_40_ of β-ecdysterone on grape phylloxera. The fourth instar and adult developmental periods and total life span were significantly prolonged by LC_40_ of β-ecdysterone. Fecundity decreased when grape phylloxera were exposed to LC_20_ and LC_40_ of β-ecdysterone. In addition, LC_40_ of β-ecdysterone decreased the intrinsic rate of increase (r_m_) and the finite rate of increase (λ) and prolonged the population doubling time (DT). The net reproductive rate (R_0_) was significantly reduced by both the LC_20_ and LC_40_ β-ecdysterone treatments. Our results demonstrated that β-ecdysterone had higher toxic effects and significant sublethal effects on grape phylloxera, and showed potential control of grape phylloxera.

## Introduction

Grape phylloxera (*Daktulosphaira vitifoliae* Fitch) is an aphid-like insect pests, native to North America [1–2], which was accidentally imported into Europe in the mid 19^th^ century [3] and nowadays is regarded as the most destructive insect pest of commercial grapevines *Vitis* spp. L. (Vitacae) worldwide [4]. Grape phylloxera is an obligate parasite of grapevines *Vitis* spp., it reproduce parthenogenetically during spring and summer on leaves and roots of susceptible vines, towards the end of the season, sexual reproduction take place due to the populations increase and the nutrient status of vines changes [5]. Phylloxera feed on leaves and roots of many grape species, forming pocket-like galls (nodosities) on leaves and hooked-like galls on root tips and root swellings (tuberosities) on mature roots. The galls on roots split and crack and feeding sites leave entry points, which allows entry of soil-borne pathogens and this can cause death of the vine [5]. Its worth noting that grapevines do not always die this is dependent on the host genotype and the insect genotype.

The common and relatively successful strategies for grape phylloxera is grafting tolerant hybrid *Vitis* spp rootstocks to the susceptible *V*. *vinifera* L. producing scions [6]. Although rootstocks have been successfully used for more than 130 years, this method is facing the risk of a breakdown in resistance via interactions between the host and pest [7]. Most of the rootstocks used nowadays are based on hybrids of North American *Vitis* species. There are relatively few reported instances of rootstock failure which occurred mainly where the parentage of a rootstock hybrid includes partial *V*. *vinifera* genetic background [7]. The emergence of “biotype B” caused a breakdown in the resistance of the widely planted rootstock AXR#1 (*V*. *vinifera* ‘Aramon’×*V*. *rupestris*) and cost the viticulture industry between 1 to 6 billion US$ [8–10].

Research on alternative and supplemental control methods are needed to back up rootstock use and prevent the losses caused by the resistant rootstocks [11]. The use of *Achyranthes bidentata* Blume has potential for grape phylloxera control [12]. *A*. *bidentata* belongs to family Amarathaceae and it has a wide application in the traditional (orthodox) and folk medicine [13]. Recently, a study showed that aqueous root extracts from *A*. *bidentata* induced mortality of grape phylloxera and that intercropping of *A*. *bidentata* and grapevines can be used to control grape phylloxera [12]. However, it is still unknown whether chemical or chemicals present in *A*. *bidentata* play an important role in controlling grape phylloxera.

In the present study, we compared the efficacy of 22 chemicals known to be in *A*. *bidentata* root extracts against grape phylloxera under laboratory conditions. We also assessed the sublethal effects of the main bioactive component on the fecundity, developmental periods and life table parameters of the grape phylloxera. Results of this study can be useful to understand the mechanism of action of *A*. *bidentata* aqueous root extracts against grape phylloxera.

## Materials and Methods

### Insects

With the authorization of Huaihua Agriculture Bureau, Hunan Province, One- to six-day-old grape phylloxera eggs were taken from five phylloxera-infested vineyards (*Vitis labruscana* Kyoho) near Shuangxi town, Huaihua city, Hunan Province, China (27°14′N, 109°51′E). The maintenance method was followed according to de Benedictis and Granett [14], After collection, fresh healthy excised root pieces (3–7 mm in diameter and 4–5 cm in length) of *Vitis labruscana* Kyoho were infested with 10–20 phylloxera eggs. One end of each root piece was wrapped in wet cotton to prevent desiccation. The infested root pieces were put into petri dishes (12-cm diameter) in controlled environment incubators (26 ± 1°C, 80 ± 5% RH, 0L:24D). The eggs ranged from one- to six-hour-old when used for inoculation.

### Chemicals

Ginsenoside Ro (98%), oleanolic acid (99%), Stigmasterol (95%), palmatine hydrochloride (98%), betherine (98%), epiberberine (98%), coptisine (98%), astragalin (98%), isoquercitrin (98%), baicalin (98%), wogonin (98%), chrysophanol (98%), physcion (98%) and Geniposide (98%) were sourced from the Chengdu Must Bio-technology Co., Ltd (Chengdu, Sichuan Province, China). Betaine (98%), nonanedioic acid (99.5%), succinic acid (90%), allantoin (98.5%), rutin (95%), β-sitosterol (95%) and 5-hydroxymethyl furaldehyde (99%) were sourced from Sinopharm Chemical Reagent Co., Ltd (Shanghai, China). β-ecdysterone was sourced from J&K Scientific Ltd (Beijing, China).

### Grapevine root-dip bioassay

A root dipping method, adopted from a leaf-dip bioassay method [15] was used to determine the toxicity of each of 22 compounds against grape phylloxera. The stock solutions (50,000 mg a.i. [active ingredient] liter^-1^) of each compound were diluted using methanol. The stock solution of 22 compounds were then further diluted with distilled water containing 0.1% Tween-80 to the desired concentrations. A total of 50 grape phylloxera eggs (approximately 6 h old) were selected from the laboratory colony and were placed on each grape root (3–7 mm in diameter and 5 cm in length) of *Vitis labruscana* Kyoho in petri dishes (12 cm diam.) which were sealed as to prevent grape phylloxera escaping or cross contamination. After the eggs hatch, root pieces with 1 d old grape phylloxera nymphs were immersed in the diluted compound solutions for about 5s and then dried on tissue paper in a fume hood for next 1.5hrs. The mortality rate of grape phylloxera treated with the 22 compounds was recorded 15 days after exposure, because of difficulties in determining grape phylloxera death or not due to their feeding characteristics of stationary. The concentration of 22 compounds was used according to the results of the preliminary tests, and preliminary tests showed that distilled water containing 0.1% Tween-80 and 2% methanol had no effects to grape phylloxera nymphs. Each treatment included 3 replicates, and each replicate was exposed to 50 grape phylloxera nymphs. The control group, which was also replicated, was treated with distilled water containing 0.1% Tween-80 and 2% methanol. For the lethal effects of β-ecdysterone, four replicates were conducted, for each treatment and control, 15–23 grape phylloxera first instar nymphs (1 d old) were immersed in the six doses (from 25 to 800 mg a.i. liter^-1^) of β-ecdysterone for 5s and then dried on tissue paper in a fume hood. The mortality rate of grape phylloxera treated with the β-ecdysterone was recorded 15 days after exposure.

### Sublethal effects of β-ecdysterone on grape phylloxera

To assess the sublethal effects of β-ecdysterone on grape phylloxera, three different concentrations LC_10_, LC_20_ and LC_40_ were used. To obtain the concentrations to be used in further experiments, the concentration-mortality regression line was first determined, the tested concentrations were then calculated from the regression lines (see “Results” section).

Root pieces with 1 d old grape phylloxera first instar nymphs were immersed in the sublethal concentration of β-ecdysterone for 5s and then dried on tissue paper in a fume hood. After drying, the roots were placed in pairs on filter paper discs in sealed glass petri dishes (12 cm diam.). One end of each root piece was wrapped in wet cotton to prevent desiccation. All of the petri dishes were maintained in controlled incubators (26 ± 1°C, 80 ± 5% RH, 0L: 24D). For each treatment and control, 200 grape phylloxera first instar nymphs were exposed to β-ecdysterone, i.e. 50 eggs were considered per replicate and four replicates per treatment and control. Bioassay plates were checked every 24 h, and the survivors after 15 d exposure were used to evaluate the following parameters: developmental duration, mortality, survival of nymphs and adult and number of eggs laid. The nymphal instar was judged by observing its ecdysis, the grape phylloxera nymphal increase instar after each ecdysis [16]. The experiments continued until the death of each individual. Life table parameters including intrinsic rate of increase (r_m_), finite rate of increase (λ), net reproductive rate (R_0_), mean generation time (T) and population doubling time (DT) were calculated.

### Data analysis

The median lethal concentrations, 95% confidence limits (CLs), and slope ± SE were calculated using probit analysis. The life table parameters with various treatments (control, β-ecdysterone LC_10_, LC_20_ and LC_40_) were calculated:

- The net reproductive rate [17–18]:
  $$R_{0}=\sum l_{x}m_{x};$$
- The intrinsic rate of increase (r_m_) was calculated according to Carey (1993) [19] and Bechmann (1994) [20], the Birch model [17] was used:
  $$\sum l_{x}m_{x}e^{\text{-}rmx}=1;$$
- The finite rate of increase [17]:
  $$\lambda =e^{rm};$$
- The mean generation time [17, 21–22]:
  $$T=lnR_{0}/r_{m};$$
- The doubling time [23]:
  $$DT=ln\left(2\right)/r_{m};$$

In the equations, l_x_ is the age-specific survival rate, which is the probability to survive to a particular age x, and m_x_ is the age-specific fecundity, which is calculated as the number of alive females per female for age x [17].

The data on developmental rate of each stage of the grape phylloxera in the various treatments and life table parameters were analyzed using a one-way ANOVA followed by Tukey's HSD (honestly significant difference) for multiple comparisons. All data were analyzed by SPSS 13.0 (SPSS Inc., Chicago). The mean mortality of grape phylloxera nymphs after treat with 22 chemicals were logit transformed before being analyzed.

## Results

### Efficacy of chemicals to grape phylloxera in laboratory

Mortality of grape phylloxera nymphs was significantly different after treatment for fifteen days (*F* = 23.60, *d*.*f*. = 22, 46, *P* < 0.001). The order of effective (high-low) for the 22 chemicals was as follows: β-ecdysterone > chrysophanol > succinic acid > oleanolic acid > stigmasterol > geniposide > β-sitosterol > coptisine > wogonin > baicalin > 5-hydroxymethyl furaldehyde > isoquercitrin > astragalin > nonanedioic acid > epiberberine > betherine > physcion > ginsenoside Ro > palmatine hydrochloride > allantoin > rutin > betaine. Among the 22 chemicals, the effective of β-ecdysterone was the highest with the mortality of 96.15±3.85% after 15 days of exposure (Table 1 and Table A in S1 Tables). Meanwhile, the betaine had the lowest effective with a mortality of only 13.10±2.69%.

**Table 1:** Mean mortality (mean±SE) of grape phylloxera nymphs at fifteen days after treat with 22 chemicals, known to occur in Achyranthes bidenta, at dose rate of 1000 mg a.i. liter^-1^.

Treatment	15 days after treatment	Treatment	15 days after treatment
**β-ecdysterone**	96.15±3.85 a	astragalin	27.08±2.50 def
**chrysophanol**	44.42±1.20 b	nonanedioic acid	25.19±4.12 bcdef
**succinic acid**	41.34±0.83 bc	epiberberine	23.03±1.84 bcdef
**oleanolic acid**	36.19±3.89 bcd	betherine	22.74±2.92 bcdef
**stigmasterol**	35.40±3.23 bcd	physcion	21.41±1.27 bcdef
**geniposide**	33.93±3.42 bcde	ginsenoside Ro	20.19±3.03 bcdef
**β-sitosterol**	31.90±3.46 bcde	palmatine hydrochloride	18.31±2.51 cdef
**coptisine**	31.34±1.47 bcde	allantoin	16.11±2.00 def
**wogonin**	30.26±1.94 bcde	rutin	15.44±1.81 def
**baicalin**	29.95±2.67 bcde	betaine	13.10±2.69 ef
**5-hydroxymethyl furaldehyde**	28.42±0.62 bcde	Control	8.65±0.83 f
**isoquercitrin**	27.26±4.68 bcde		

Notes: Means followed by the same letters are not significantly different at *P*>0.05 (Tukey’s HSD test).

### Lethal effects of β-ecdysterone

**Fig 1:**
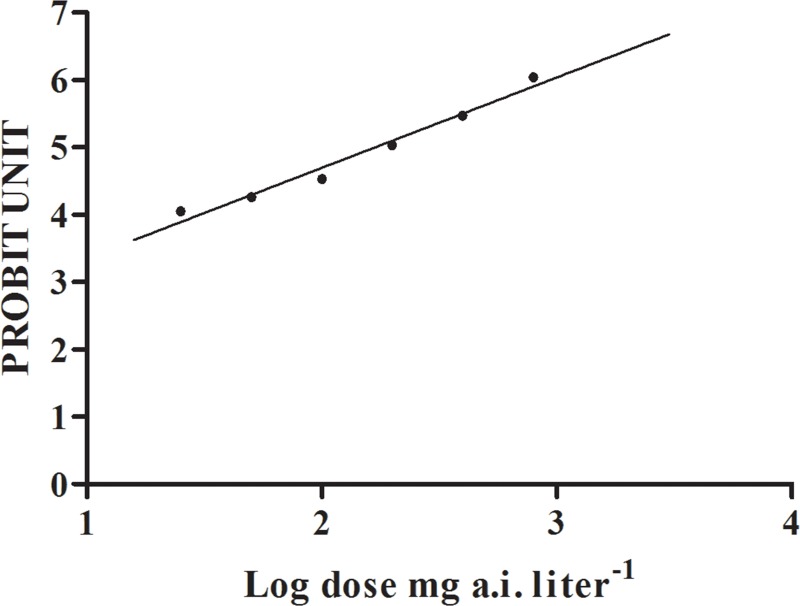
Linear regression between morality (probit unit) of grape phylloxera and β-ecdysterone concentration (log-transformed).

The linear regression of dose-mortality relationship was fitted to the actual data for β-ecdysterone tested. The LC_50_ value of β-ecdysterone was considered valid since there was no significant deviation between the observed and the expected data (Fig 1 and Table B in S1 Tables). The LC_50_ value of β-ecdysterone against grape phylloxera at 15d was 175.73 mg a.i. liter^-1^ (*Slope* = 1.35, *SE* = 0.14, χ^2^ = 16.75, *df* = 22, *P* = 0.777). Estimated LC_40_, LC_20_, LC_10_ β-ecdysterone values were 113.99, 41.72 and 19.67 mg a.i. liter^-1^, respectively.

### Sublethal effects of β-ecdysterone on developmental period of grape phylloxera

The mortality tests after 15d of exposure were 2.7%, 8.6%, 23.5% and 43.6% for control, LC_10_, LC_20_ and LC_40_ groups, recorded respectively. These three doses could not be considered as sublethal doses but could induce multiple sublethal effects in exposed individuals (according to Desneux et al. [21]).

The results presented in Table 2 show the effect of various β-ecdysterone treatments on measured life history parameters of grape phylloxera (Table C in S1 Tables).

**Table 2:** Life history parameters of grape phylloxera treated by β-ecdysterone at three lethal concentrations.

Treatments	Development (days)							Fecundity (eggs)
	Egg incubation	Nymphs				adults	Total life span	
		1^st^ instar	2^nd^ instar	3^rd^ instar	4^th^ instar			
**LC**_**10**_	5.677 ± 0.055 a	11.142 ± 0.748 a	2.013 ± 0.095 a	1.798 ± 0.065 a	1.369 ± 0.006 ab	26.493 ± 0.506 a	48.518 ± 1.283 a	211.868 ± 3.512 a
**LC**_**20**_	5.677 ± 0.091 a	11.427 ± 0.496 a	2.016 ± 0.036 a	1.762 ± 0.064 a	1.450 ± 0.029 ab	24.865 ± 1.521 a	45.948 ± 0.591 a	185.319 ± 3.013 b
**LC**_**40**_	5.688 ± 0.078 a	11.607 ± 0.415 a	1.994 ± 0.082 a	1.710 ± 0.127 a	1.513 ± 0.029 b	15.233 ± 0.335 b	37.745 ± 0.635 b	111.178 ± 2.838 c
**Control**	5.617 ± 0.077 a	11.947 ± 0.928 a	1.998 ± 0.063 a	1.724 ± 0.076 a	1.341 ± 0.066 a	27.498 ± 0.747 a	50.110 ± 1.410 a	219.978 ± 2.938 a

The data in the table are mean ± SE, and those in the same column followed by same letters are not significantly different at *P*<0.05 (Tukey’s HSD test).

LC_10_, LC_20_ and LC_40_ of β-ecdysterone had no significant effects on first (*F* = 0.25, *d*.*f*. = 3, 12, *P* = 0.86), second (*F* = 0.021, *d*.*f*. = 3, 12, *P* = 1.00) and third (*F* = 0.22, *d*.*f*. = 3, 12, *P* = 0.88) instars developmental periods. Both the fourth instar (*F* = 39.15, *d*.*f*. = 3, 12, *P* < 0.001) and adult (*F* = 27.56, *d*.*f*. = 3, 12, *P* < 0.001) developmental period and total life span (*F* = 257.64, *d*.*f*. = 3, 12, *P* < 0.001) were significantly prolonged by LC_40_ of β-ecdysterone, whereas LC_10_ and LC_20_ treatments did not significantly affect fourth instar and adult developmental period and total life span of grape phylloxera. Grape phylloxera fecundity was significantly reduced by both the LC_20_ and LC_40_ β-ecdysterone treatments compared to the control (*F* = 257.64, *d*.*f*. = 3, 12, *P* < 0.001) and LC_10_ treatment. The fecundity of grape phylloxera decreased with increasing doses of β-ecdysterone significantly.

### Sublethal effects of β-ecdysterone on life table parameters of grape phylloxera

Table 3 data shows the life table parameters of grape phylloxera treated with β-ecdysterone (Table D in S1 Tables).

**Table 3:** Life table parameters of grape phylloxera treated by β-ecdysterone three lethal concentrations.

Treatments	Intrinsic rate of increase (r_m_)	Finite rate of increase (λ)	Net reproductive rate (R_0_)	Mean generation time (T)	Population doubling time (DT)
LC_10_	0.171 ± 0.005 a	1.186 ± 0.006 a	203.822 ±3.101 a	31.178 ± 0.854 a	4.066 ± 0.122 a
LC_20_	0.164 ± 0.003 ab	1.179 ± 0.003 ab	177.860 ± 4.786 b	31.550 ± 0.368 a	4.223 ± 0.068 ab
LC_40_	0.150 ± 0.002 b	1.163 ± 0.003 b	102.461 ± 3.362 c	30.416 ± 0.777 a	4.595 ± 0.072 b
Control	0.167 ± 0.005 ab	1.182 ± 0.006 ab	216.139 ± 3.659 a	32.276 ± 1.042 a	4.161 ± 0.129 a

The data in the table are mean ± SE, and those in the same column followed by same letters are not significantly different at *P*<0.05 (Tukey’s HSD test).

Compared to the LC_10_ of β-ecdysterone, the exposure to LC_40_ of β-ecdysterone significantly reduced the intrinsic rate of increase ‘r_m_’ (*F* = 4.46, *d*.*f*. = 3, 12, *P* = 0.025) and the finite rate of increase ‘λ’ (*F* = 4.40, *d*.*f*. = 3, 12, *P* = 0.026), which decreased with the exposure of β-ecdysterone increase dose, and there was no difference between the control and each of the three β-ecdysterone concentrations. Net reproductive rate ‘R_0_’ was significantly reduced by LC_20_ and LC_40_ β-ecdysterone treatments compared to the control (*F* = 257.64, *d*.*f*. = 3, 12, *P* < 0.001), and the ‘R_0_’ decreased with increasing doses of β-ecdysterone from LC_10_ to LC_40_. No significant difference was found in mean generation time ‘T’ between β-ecdysterone different treatments and control (*F* = 0.94, *d*.*f*. = 3, 12, *P* < 0.45). The population doubling time ‘DT’ was significantly prolonged by LC_40_ of β-ecdysterone compared to the control, and showed a downward trend.

## Discussion

*Achyranthes bidentata* is widely distributed in China, Korea, and Vietnam [13]. Meng (2004) identified 34 compounds from *A*. *bidentata* by physico-chemical characteristics and spectroscopic analysis [24]. Others including five phenolic compounds, seven triterpenoid saponins, betaine, Stigmasterol, Chrysophanol and allantoin were identified by Nicolov *et al*.(1996), Li *et al*.(2007), Zhao *et al*. (2011), Hu *et al*. (2004), Wei *et al*. (1997) and Chao *et al*. (1999), respectively [13, 25–29]. In the present study, 22 of these compounds, which were easily synthesized and readily available, were selected for use in the laboratory bioassay and overall β-ecdysterone showed the highest toxic effect towards grape phylloxera. However the other compounds tested which are also known to be present in and *A*. *bidentata* root extracts possess insecticidal activity against grape phylloxera need further research to be conducted.

Ecdysteroids belong to a very large group of cholesterol-derived molecules which also comprise plant hormones [30], plant secondary metabolites and many polyhydroxysterols [31]. The discovery of ecdysteroids in plants [32–34] resulted in the commercial availability of ecdysteroids to all insect physiologists, because they can regulate insect growth, development and reproduction [35]. Our results showed that β-ecdysterone have significant lethal and sublethal effects on grape phylloxera, this may be related to the toxic characteristics of β-ecdysterone, such as antifeedant, growth and development inhibitive activities [36–37].

The sublethal effects of β-ecdysterone on grape phylloxera demonstrated that an LC_40_ of β-ecdysterone increased fourth instar development time, this may be related to the delay in the time of ecdysis. Because ecdysteroid can cause a delay in the time of ecdysis [38] and β-ecdysterone has the same structure as the ecdysteroid secreted by insect or other arthropods [35]. But this phenomenon did not find in other instar, therefore, it still needs a further research. Moreover, the sublethal effects of β-ecdysterone on grape phylloxera demonstrated that an LC_40_ of β-ecdysterone decreased adult development time and fecundity. The reason may be that a certain dose of β-ecdysterone can cause insect antifeedant or stop feeding [39–40]. The behaviors of antifeeding and stop feeding can cause inadequate intake of nutrients, coupled with the metabolism and degradation reactions on the consumption of nutrients, resulting in reduced of nutrients content, thereby causing decrease in adult longevity and fecundity.

The intrinsic rate of increase (r_m_) is a measure of the ability of a population to increase exponentially in an unlimited environment. It provides an effective summary of an insect’s life history traits[41] and has also been recommended together with toxicity assessment to provide a more accurate estimate of population-level effect of toxic compounds [42–44]. In our study, the exposure to an LC_40_ of β-ecdysterone significantly reduced the intrinsic rate of ‘r_m_’, which decreased with the exposure to increasing doses of β-ecdysterone. This means that population increase of grape phylloxera was delayed when using an LC_40_ β-ecdysterone treatment.

Many studies have shown that sublethal doses exert devastating effects on insects by increasing the development time [28, 45–47], reducing fecundity [48–50] and decreasing egg hatching rate [51]. However, sublethal effects sometimes also show positive impacts on the insects [52, 53]. Previous studies showed that low concentrations of imidacloprid increased the biological fitness of green peach aphid *Myzus persicae* (Sulzer) [54], prolonged the nymph development of whitefly *Bemisia tabaci* (Gennadius) [55] and enhanced the fecundity of spider mite *Tetranychus urticae* Koch [56]. In this study, we found that at the three low concentrations of β-ecdysterone had no positive impacts on grape phylloxera and still can reduce their population.

Our study showed that β-ecdysterone may be the main bioactive component of *A*. *bidentata* against grape phylloxera, and it has potential for the control of grape phylloxera. However, considering the limited number of compounds, a suite of compounds require further testing. Meanwhile, the control effects to grape phylloxera by intercropping *A*. *bidentata* and grapevines caused by β-ecdysterone released from *A*. *bidentata* need a further research.

## Supporting Information

S1 Tables Spreadsheet tables presenting supplementary data. (XLSX)
